# Meetings of Societies

**Published:** 1926

**Authors:** 


### Meetings of Societies.

### Bristol Medico-Chirurgical Society.

The Annual Meeting of the Society was held in the University on Wednesday, October 14th, 1925.

After a vote of thanks to the retiring President, Dr. J. O. Symes, had been enthusiastically carried, Mr. T. Carwardine took the chair, and gave an introductory address on "**Continuity and Change in Life.**" Later a cordial vote of thanks was given to Mr. Carwardine for the able and interesting address he had delivered.

The Hon. Secretary, Mr. S. V. Stock, and the Editorial Secretary read their annual reports.

The following are the officers of the Society for the Session 1925–26 : *President*—Mr. T. Carwardine ; *President-Elect*—Mr. A. L. Flemming. *Committee* (*ex-officio*)—Dr. J. O. Symes ; Dr. Patrick Watson-Williams (*Editor*) ; Dr. C. King Rudge (*Hon. Medical Librarian*) ; Mr. A. L. Flemming (*Editorial Secretary*) ; Mr. S. V. Stock (*Hon. Sec.*). *Elected*—Mr. Lace, Dr. Nixon, Mr. Rayner, Mr. C. F. Walters, Mr. Hey Groves, Mr. Wright, Dr. H. L. Ormerod, Dr. Vincent Wood.

Dr. Parker and Dr. W. A. Smith represent the Society on the University Library Sub-Committee.

### INCOME AND EXPENDITURE ACCOUNT OF THE BRISTOL MEDICO-CHIRURGICAL SOCIETY, 1924-1925.

**Receipts.**

| | £ | s. | d. |
|---|---|---|---|
| Balance brought forward from last Account— | | | |
| Cash at Bank— | | | |
| Current Account .. | 68 | 6 | 2 |
| Deposit Account .. | 117 | 2 | 2 |
| Cash in hands of Hon. Sec. .. .. .. .. .. | 1 | 9 | 9 |
| Members' Subscriptions .. | 106 | 9 | 6 |
| Interest on Bank Deposit .. | 2 | 11 | 2 |
| | £295 | 18 | 9 |

**Payments.**

| | | | | £ | s. | d. |
|---|---|---|---|---|---|---|
| To *Bristol Medico-Chirurgical Journal* .. .. .. .. .. | | | | 175 | 0 | 0 |
| Librarian Honorarium .. | | | | 15 | 0 | 0 |
| University Marshal .. | | | | 1 | 0 | 0 |
| Miscellaneous Expenses— | | | | | | |
| Postages and Sundries .. | £7 | 8 | 0 | | | |
| Printing and Stationery | 4 | 0 | 6 | | | |
| Lantern at Meetings .. | 3 | 3 | 0 | | | |
| Subscription Lewis Library .. .. | 5 | 1 | 0 | | | |
| Repairs, Furniture .. .. | 14 | 3 | 6 | | | |
| Do., Pictures .. | 2 | 8 | 0 | | | |
| | | | | 36 | 4 | 0 |
| Forward to next Account— | | | | | | |
| Cash at Bank— | | | | | | |
| Current A/c | 46 | 2 | 2 | | | |
| Deposit A/c | 19 | 13 | 4 | | | |
| Cash in hands of Hon. Sec. | 2 | 19 | 3 | | | |
| | | | | 68 | 14 | 9 |
| | | | | £295 | 18 | 9 |

*Wednesday, November 11th,* 1925.

Mr. T. Carwardine, President, in the Chair.

Dr. Carleton read a paper on "**Some Problems of Pulmonary Disease with Special Reference to Radiography,**" reported with the ensuing discussion in our last number.

---

*December 9th,* 1925.

At this meeting the Society were the hosts of the Long Fox Lecturer, Dr. Carey Coombs. The lecture is reported on page 1 of this issue.

*January* 13*th*, 1926.

MR. T. CARWARDINE, President, in the Chair.

DR. ELWIN HARRIS exhibited a specimen of a **Calcified Fibroid.** On removal, this was cylindrical in shape, rounded at each end, two and a half inches in length, and about the same in circumference. It was encased in plates of calcareous material, on which were excrescences, some of which were as sharp as needles. The interior had also undergone calcareous degeneration. It was removed from a married lady of 38. She had one child only, who is now eleven years old. Since 20 years of age she suffered from indefinite pains in the pelvis and lower part of the abdomen. When 22 she was operated upon for an acute attack of appendicitis, and although she made a good recovery, the pains continued. Four years later she became pregnant. This ran its ordinary course, and there was no difficulty at parturition, but afterwards the pains were worse than ever. There was no menorrhagia. Dr. Harris examined her for the first time in October last, and found a hard swelling occupying Douglas' pouch, which was attached to the posterior surface of the uterus. On examining per rectum, a number of excrescences on the surface of the tumour were felt, so exceptionally sharp that, with moderate pressure, one could have easily injured the rectum. To leave a tumour of this nature in the peritoneal cavity would have been dangerous, and on November 26th Dr. Harris removed the tumour, which was found in Douglas' pouch, not in any way forming part of the uterus, but simply connected with it by a broad, flat adhesion; there were also adhesions to the brim of the pelvis, the omentum, and the small intestine. Removal was easy. The patient made an excellent recovery, and has had no return of the pains. Calcification of fibroids is generally found in old people. In this case the patient was comparatively young, and this occurrence may be explained as follows: this tumour started life as a subperitoneal fibroid with a pedicle. During the attack of appendicitis some adhesion probably formed which more or less anchored it in the pelvis. Pregnancy then commenced, and as the uterus rose higher and higher the pedicle was more and more stretched until eventually it snapped. The fibroid, deprived of its main source of blood supply, degenerated, calcified, and the sharp excrescences, in course of time, gave rise to the adhesions found at the operation.

MR. E. WATSON-WILLIAMS showed two cases of **Carcinoma of the Vocal Cord in Men.**

CASE 1.—Following laryngofissure in February, 1923, the first patient had remained perfectly well (three years). He is out daily, working as a newsvendor, and does about ten miles in his round. There is no sign of recurrence ; several bands of scar tissue in part replace the vocal cord. As the arytenoid had to be removed, these bands are immobile ; the voice is hoarse, but fairly strong.

CASE 2.—A man of 52 had been under treatment for six months for genital tuberculosis. Although hoarse for a time before, and very hoarse since July, 1925, his laryngitis had been supposed to be tuberculous. However, on examination in October a typically cancerous growth of the nodular type was seen, and its nature confirmed by biopsy. Operation was refused. This case afforded an interesting example of the importance of *always* examining the larynx in a patient with persistent (and especially painless) hoarseness.

DR. S. HARDY KINGSTON showed the following cases :—

CASE 1.—**Palmar Syphilide.** P.T., married, aged 40. Note the triangle of one palm only affected, presenting sheets of thickened exfoliating skin over a raw, inflamed area. This, associated with the dull red or purple nodules on opposite forearm, as well as the rupial condition of one knee, completes a clinical picture confirmed by + + Wassermann and history of miscarriages. To be diagnosed from (1) Tinea of the palm. Scales usually smaller and exhibit fungus under the microscope. (2) Psoriasis. Eruption would be generalised and affecting the extensor aspect generally. (3) Seborr. Eczema. Condition bilateral and involving whole hand.

CASE 2.—**Lupus Vulgaris associated with Rodent Tumour.** Patient, a male, aged 60. Has had lupus of face for forty years. The pale scarring is very evident as the result of extensive ulceration. A raised pearly-looking tumour, half an inch in diameter, situated on the upper and posterior edge of the scar, proved by section to be a rodent tumour. This combination is unique.

CASE 3.—**Exfoliative Dermatitis (Arsenical),** following the exhibition of four doses of N.A.B. Note the generalised scaling from head to foot surmounting a profound erythema, and associated with intense pruritus. The soles and palms show hyperkeratosis; the finger-nails are undergoing a process of fissuring and destruction. A most obstinate feature has been the hyperidrosis. Sodium thiosulphate was a failure in this case. This is not generally so. He is improving under intramuscular injection of contramine—a sulphur derivative.

CASE 4.—**Acne Rosacea.** Female, aged 30. This case illustrates the oily condition of the skin with inflamed pilosebaceous follicles leading to the formation of acne-like lesions. This patient, who was first seen yesterday, is shown to contrast with Mrs. X., aged 40, who has responded to treatment on the following lines: (1) Shampoo scalp once every ten days and apply each morning an antiseptic spirit lotion of sulphur or resorcin or mercury and salicylic acid. (2) Steam the face every night for ten or fifteen minutes, followed by the application of a lotion such as sulphur and chalk.

CASE 5.—**Lupus Erythematosus.** Female, aged 26. Showing typical "bat's-wing area" of face occupied by pink patches with raised infiltrated borders replaced in some areas by thin white scars, indicating retrogressive changes of an atrophic character. The ears show definite depressed cicatrizes, the result of superficial ulceration.

CASE 6.—**Lupus Erythematosus.** Female, aged 50. Duration of disease five years. Shown to illustrate the involvement of the scalp with its scaling pink atrophic area as large as a saucer. Note.—Lupus vulgaris never attacks the scalp.

The fifth case is responding to treatment.

The sixth has progressed in spite of treatment.

MR. E. R. CHAMBERS read a paper on "**The Ocular Signs of Migraine,**" reported with the ensuing discussion on page 29.

### British Medical Association.

(BATH AND BRISTOL BRANCH.)

At the Clinical Meeting of the Branch held in the Medical Library of the University of Bristol on Wednesday, January 27th, the flag which the Bristol Division is presenting to the British Medical Association to be hung in the Great Hall of the New Building was on view.

The following cases and specimens were on the programme :—

MR. A. W. ADAMS : Traumatic tetany ; tumour of the right intercarotid body.

DR. CHARLES : Spinal lesion showing Brown-Séquard syndrome ; manganese poisoning.

MR. H. CHITTY : Osteogenesis imperfecta.

DR. R. C. CLARKE : Congenital syphilis ; congenital heart.

MR. J. P. I. HARTY : Postcricoid carcinoma, operation ; nasopharyngeal fibroma, operation and recurrence ; carcinoma of the pharynx ; tertiary specific laryngitis.

DR. HERAPATH : Congenital heart.

MR. A. E. ILES : Juvenile tabes with optic atrophy.

DR. S. HARDY KINGSTON : Leprosy and photographs of a second case ; lupus erythematosus, two cases ; lupus vulgaris, three cases ; acne rosacea, three cases ; exfoliative dermatitis ; arsenical dermatitis ; trade dermatitis ; multiple tumours of body (? diagnosis).

DR. MUNDY : Dislocation of 1st cervical vertebra.

DR. PHILLIPS : Myoclonic contractions of diaphragm after lethargic encephalitis.

MR. A. R. SHORT : Osteitis fibrosa of the clavicle treated by rib graft.

DR. J. O. SYMES : Hæmochromatosis.

DR. TODD : Chronic anterior poliomyelitis ; spina bifida occulta ; subacute lymphatic granulomatosis.

DR. KENNETH WILLS : Lupus vulgaris treated with Pyrotropine ; toxic dermatitis treated with Light ; ulcer of nasal bridge resembling rodent ulcer.

MR. DUNCAN WOOD : Abscess of radius ; splenomegaly.

MR. ADAMS and MR. CHAMBERS : Tumour of lacrimal gland, Kronlein approach.

DR. NIXON and MR. CHITTY : X-rays showing cholecystitis in diabetes from injections of tetra-iodo-phenolphthalein sodium.

DR. NIXON and MR. WALTERS : A case showing the result of Steinach's operation.

MR. WALTERS and DR. HERAPATH : Acholuric jaundice and notes of a second case.

MR. CHITTY and DR. HERAPATH : Renal dwarfism showing rickets.

DR. BRISTOWE : Two Neolithic skulls.

DR. CLARKE : Skull with Parrot's nodes.

DR. FRASER and DR. HADFIELD showed some pathological specimens.

